# Supplementary material for: Mechanistic insights into the activity of SARS-CoV-2 RNA polymerase inhibitors using single-molecule FRET
Source: Nucleic Acids Res. 2025 Apr 29;53(8):gkaf351. doi: 10.1093/nar/gkaf351 (PMC12038395; doi:10.1093/nar/gkaf351)
Supplement: gkaf351_Supplemental_File [file gkaf351_supplemental_file.docx]

**Supplementary Figures and Tables**

**Mechanistic insights into the activity of SARS-CoV-2 RNA polymerase inhibitors using single-molecule FRET**

Danielle Groves^1^, Rory Cunnison^1^, Andrew McMahon^1^, Haitian Fan^2^, Jane Sharps^2^, Adrian Deng^3^, Jeremy R. Keown^3^, Ervin Fodor^2^ & Nicole C. Robb^1*^

^1^Warwick Medical School, University of Warwick, Coventry, CV4 7AL, UK

^2^Sir William Dunn School of Pathology, University of Oxford, Oxford, OX1 3PU, UK

^3^School of Life Sciences, University of Warwick, Coventry, CV4 7AL, UK

*Nicole.Robb@warwick.ac.uk

| **RNA** | **Sequence** |
| --- | --- |
| Template | 5' - /Cy3/UUUUUUUUUUAAUUCUUAAUCUCACAUAGC - 3' |
| Primer | 5' - /Cy5/GCUAUGUGAGAUUAAGAAUU - 3' |
| Template swapped | 5' - /ATTO647N/UUUUUUUUUUAAUUCUUAAUCUCACAUAGC - 3' |
| Primer swapped | 5' - /Cy3/GCUAUGUGAGAUUAAGAAUU - 3' |
| Extension Template P1 | 5' - /Cy3/AAAAAAAAUUUUUUUUUUUUUUUAAUUCUUAAUCUCACAUAGC - 3' |
| Extension Primer P12 long | 5' - GCUAUGUGAGAU/Cy5/UAAGAAUUAAAAAAAAAAAAAAAUUUUUUUU - 3' |
| Extension Primer P12 short | 5' - GCUAUGUGAGAU/Cy5/UAAGAAUU - 3' |
| Unlabelled Template | 5' - UUUUUUUUUUAAUUCUUAAUCUCACAUAGC - 3' |
| Unlabelled Primer | 5' - GCUAUGUGAGAUUAAGAAUU - 3' |
| Unlabelled extension Template | 5' - AAAAAAAAUUUUUUUUUUUUUUUAAUUCUUAAUCUCACAUAGC - 3' |
| Template Cy3 P3 | 5' - CCA/Cy3/AAAAAAAUUUUUGGUUUAAUUCUUAAUCUCACAUAGC - 3' |
| Primer Cy5 P19 | 5' - GCUAUGUGAGAUUAAGAAU/Cy5/U - 3' |

**Supplementary Table 1.** Sequences and fluorescence dye positions of the RNAs used in the study. /Cy3/: position of the Cy3 label, /Cy5/: position of the Cy5 label, /ATTO647/: position of the ATTO647 label.

**
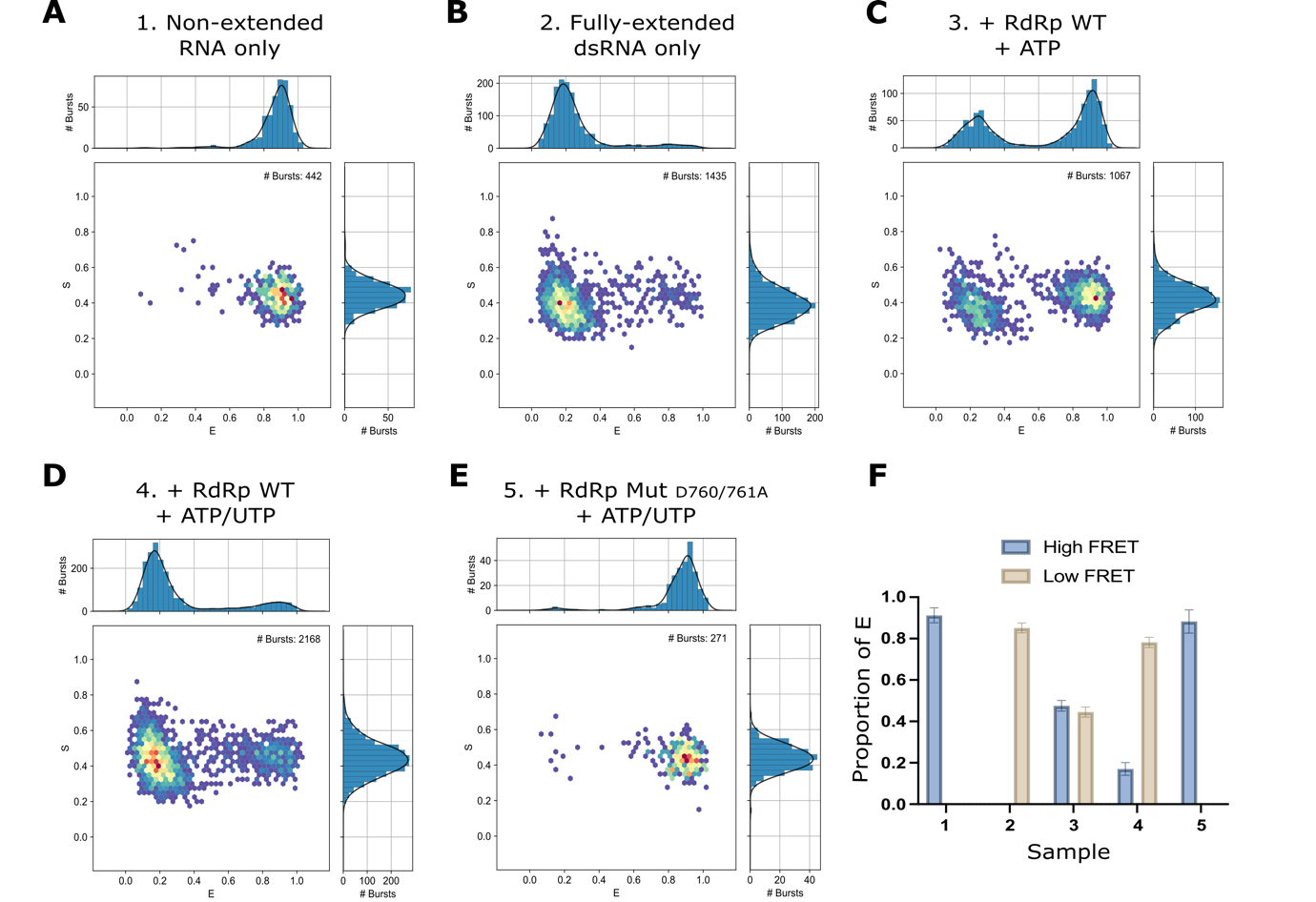
**

**Supplementary Figure 1. Single-molecule FRET can be used to measure RNA extension by the SARS-CoV-2 RdRp.** A-E) Single-molecule E-S histograms from the data presented in Fig 3. E represents apparent FRET efficiency and S represents stoichiometry. F) Quantification of the high and low FRET populations, distinguished by Gaussian fits as a proportion of the total bursts, where samples 1-5 refer to panels A-E, respectively.

**
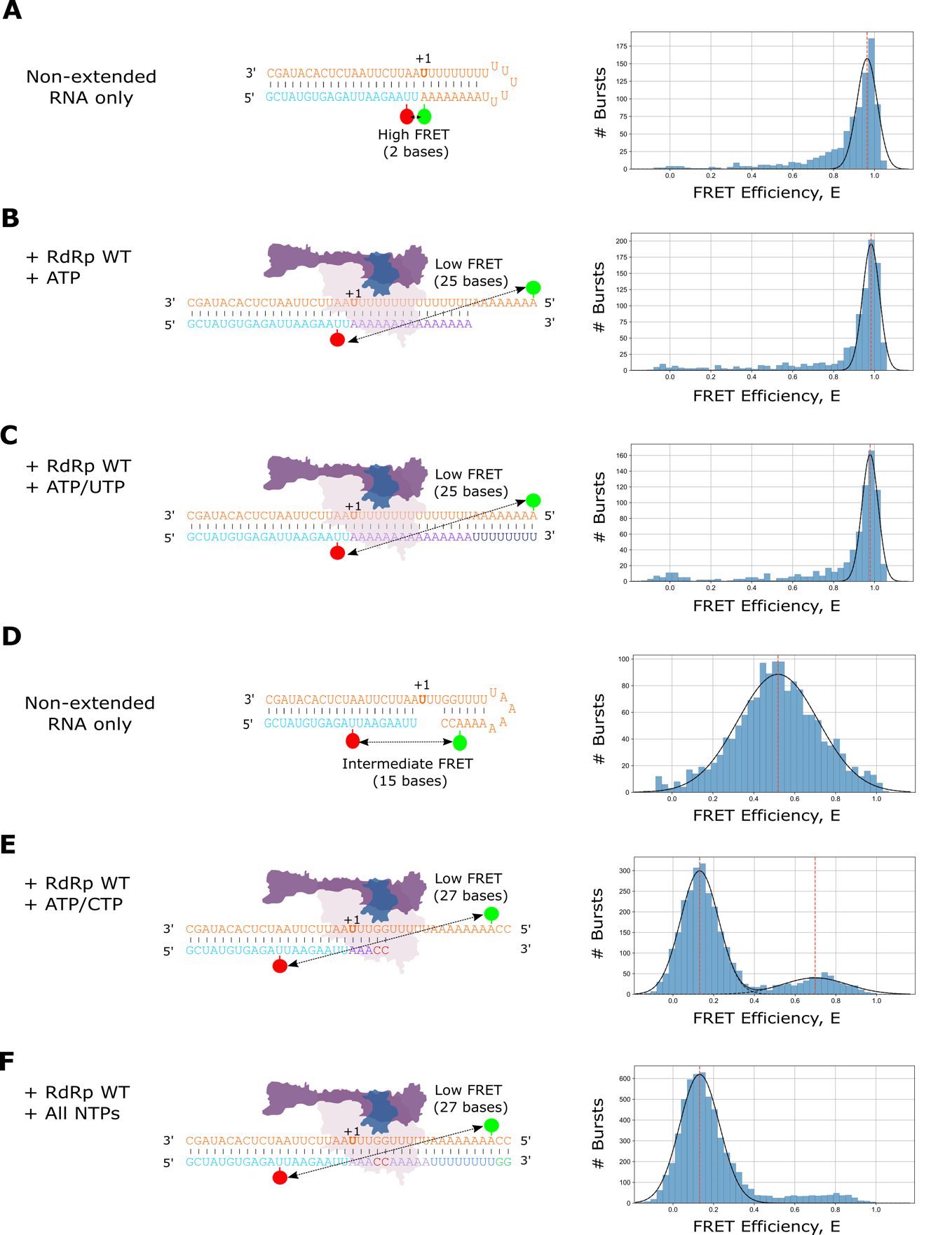
**

**
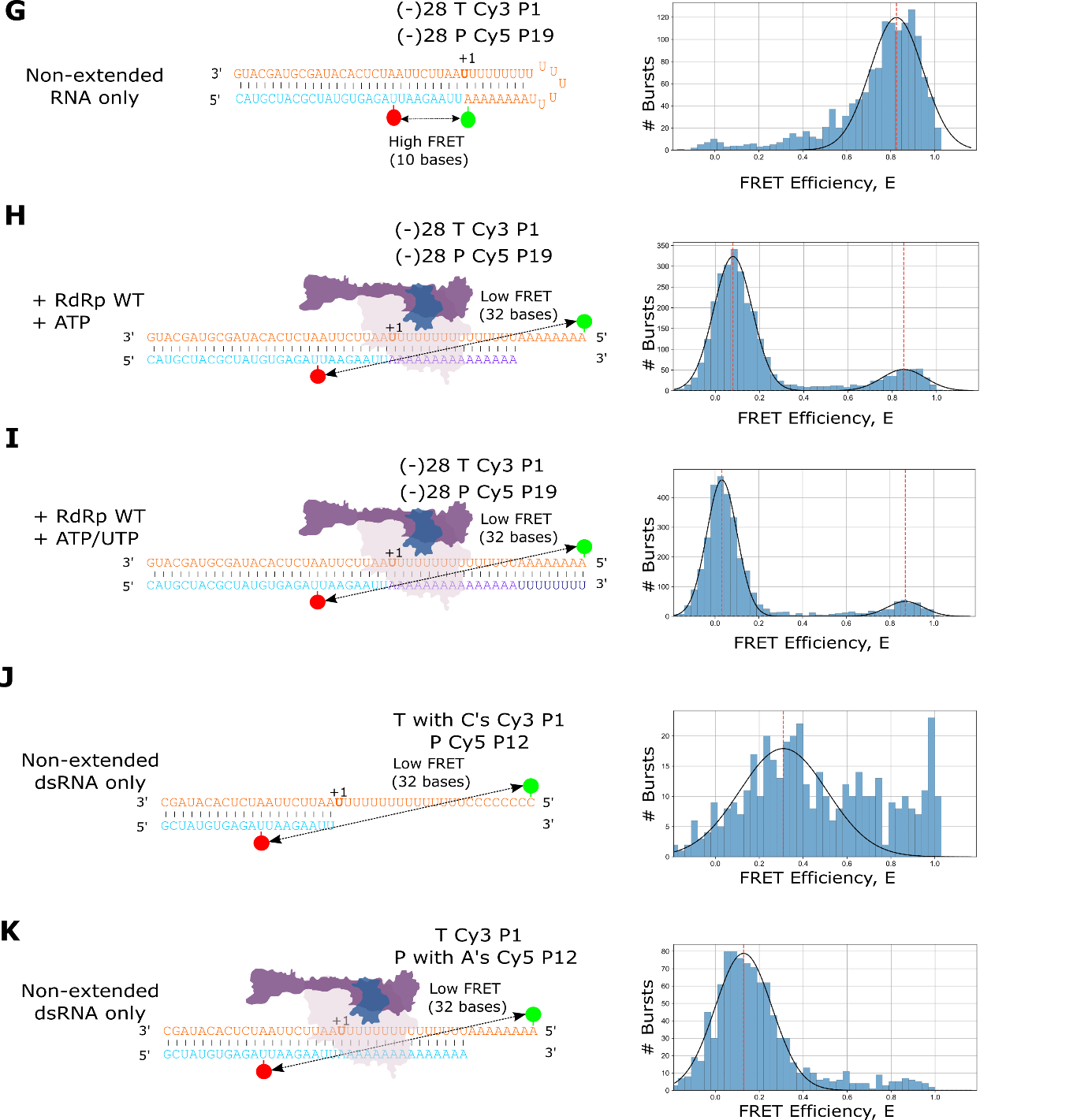
**

**Supplementary Figure 2. Investigation of alternative labelling positions for the single-molecule FRET extension assay.** A) FRET efficiency (E) for RNA only when the Cy5 dye on the 20mer primer was moved to position 19. B) Addition of RdRp and ATP to the pre-annealed RNA resulted in no extension of the primer, suggesting that the dye at position 19 is inhibitory. C) Addition of RdRp and ATP/UTP also resulted in no extension of the primer. D) FRET efficiency (E) for RNA only when the Cy3 dye on a shortened template was moved to position 3. E) Addition of RdRp and ATP/CTP to the pre-annealed RNA resulted in a shift to low FRET, suggesting opening of the template RNA. F) Addition of RdRp and all NTPS to the pre-annealed RNA provided similar results to E). G) FRET efficiency for RNA only when the Cy5 dye was placed on a longer 28mer primer. H) Addition of RdRp and ATP to the pre-annealed 53mer template and 28mer primer RNA resulted in primer extension and hairpin opening. I) Addition of RdRp and ATP/UTP also resulted in full extension of the primer and hairpin opening. J) FRET efficiency for RNA only when the template was mutated so it was unable to form a hairpin. K) FRET efficiency for RNA only when the primer included a poly(A) extension to mimic the partially extended state.

**
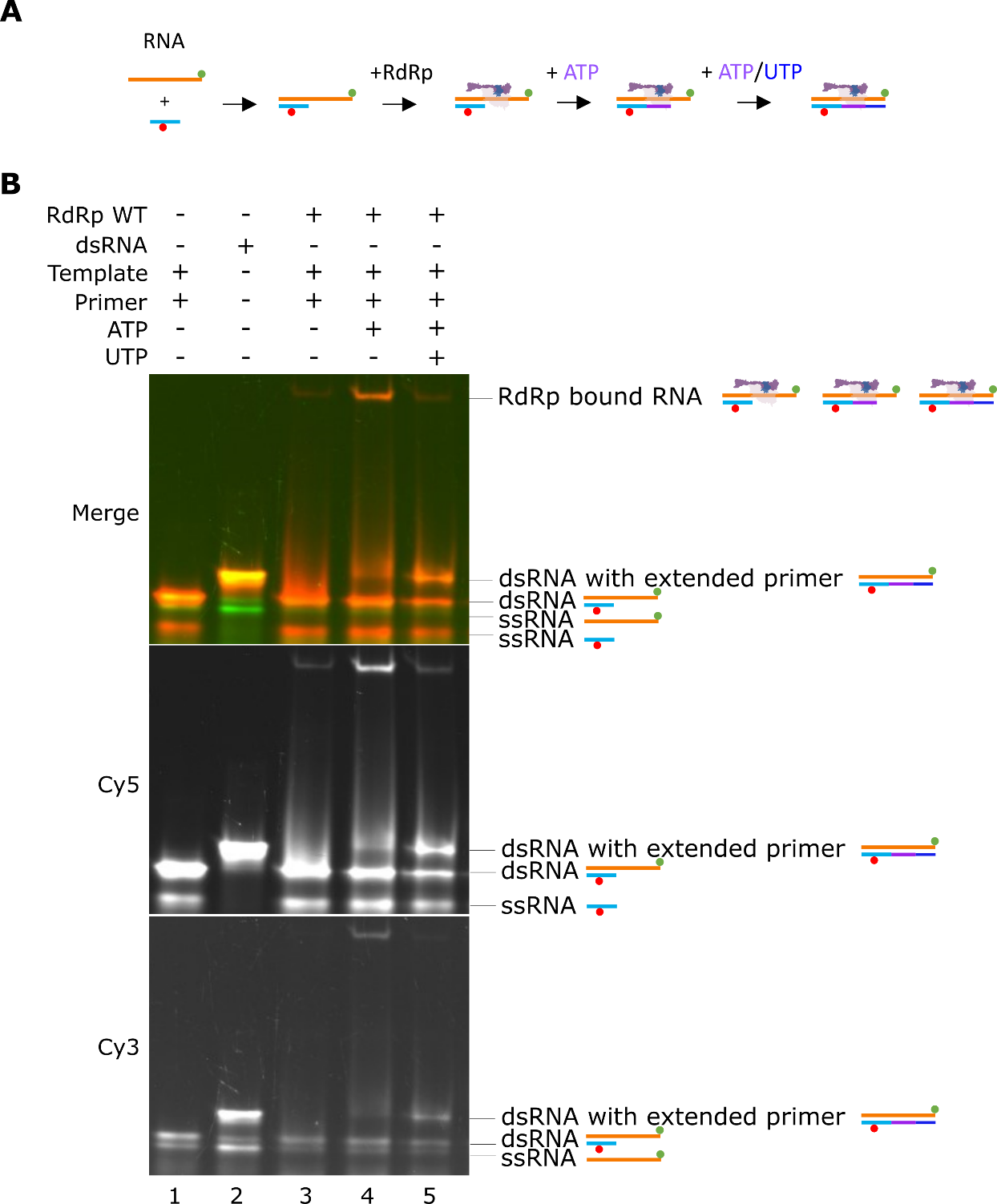
**

**Supplementary Figure 3. Native gel electrophoresis shows RdRp-RNA complexes during replication.** A) Schematic of RdRp binding to the fluorescently labelled RNA during sequential NTP addition B) Native gel showing binding of RdRp to the extension RNA. Labelled primer and template RNA run as two distinct single-stranded RNA bands as well as a double-stranded RNA (lane 1). A double-stranded RNA control that mimics the expected product predicted from full extension of the primer gives the position of the fully extended RNA (lane 2). Addition of RdRp alone does not result in primer extension but does show an RdRp-bound band higher in the gel (lane 3). Addition of RdRp and ATP results in enhanced stalling of RdRp on the partially extended primer substrate (lane 4), whilst addition of ATP and UTP results in an increase in run-off of the RdRp and an increase in the double-stranded RNA product (lane 5).


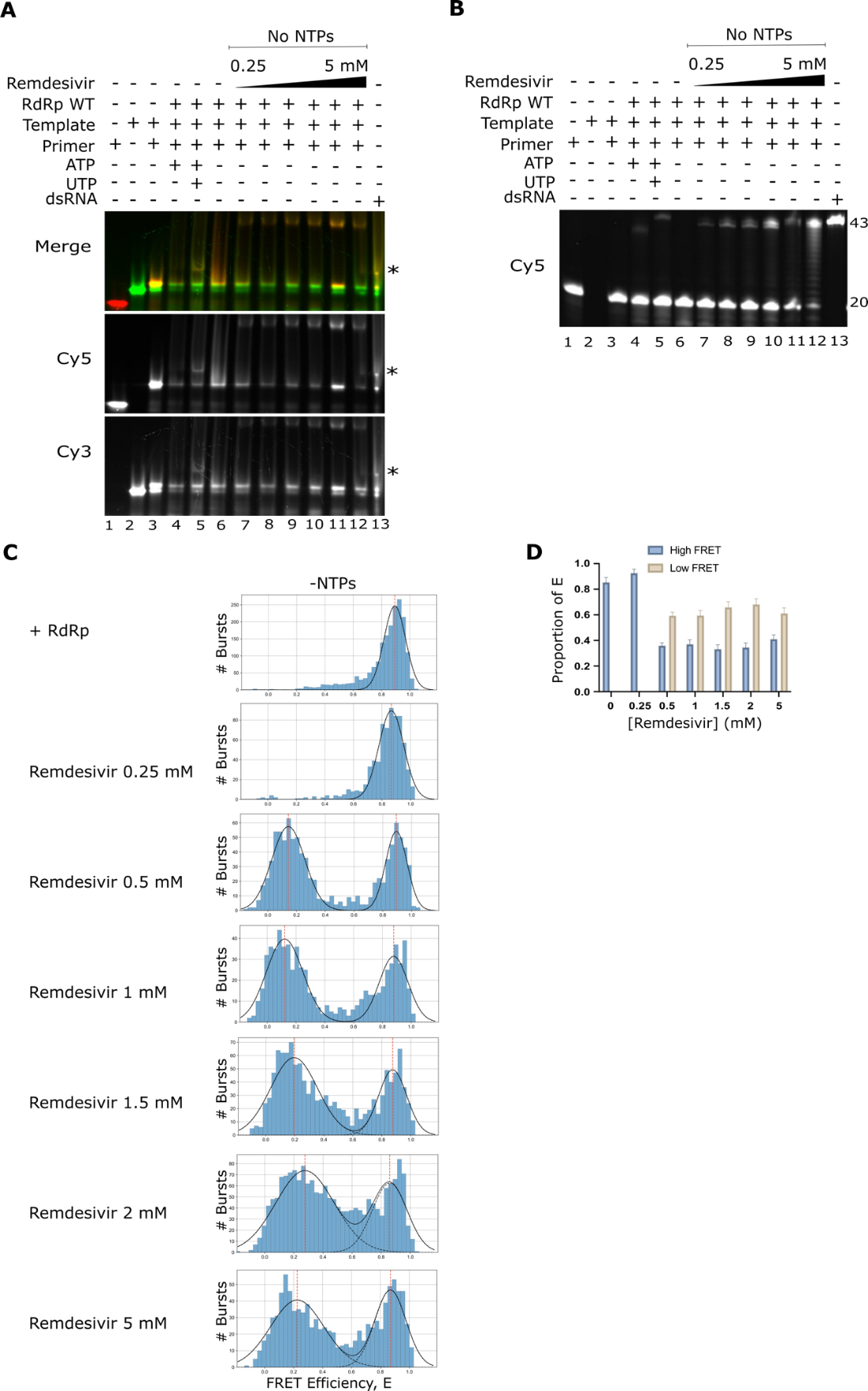


**Supplementary Figure 4. Inhibition of RNA extension with remdesivir and no NTPs.** A) Native gel showing binding of RdRp to the extension RNA. Labelled primer and template RNA run as two distinct single-stranded RNA bands as well as a double-stranded, non-extended RNA (Lanes 1-3). Addition of RdRp and both ATP and UTP are required to extend the RNA to a fully double-stranded RNA, denoted by * (Lane 5). Addition of RdRp and remdesivir without any natural NTPs present results in a shifted RdRp-bound band (Lanes 7-12). A double-stranded RNA control that mimics the expected product predicted from full extension of the primer gives the position of the fully extended RNA, denoted by * (Lane 13). B) Denaturing gel showing RdRp-mediated extension of a Cy5-labelled primer in the presence of increasing concentrations of remdesivir. C) FRET efficiencies, E, for RNA conformations during extension by the WT RdRp with increasing concentrations of remdesivir. D) Quantification of the high and low FRET populations in C) as a proportion of the total FRET distributions by finding the probability density function. Error bars represent standard error of the gaussian fit.
